# A Hospital-Wide Open-Label Cluster Crossover Pragmatic Comparative Effectiveness Randomized Trial Comparing Normal Saline to Ringer’s Lactate: Protocol and Statistical Analysis Plan of The FLUID Trial

**DOI:** 10.2196/51783

**Published:** 2023-10-06

**Authors:** Julia F Shaw, Yongdong Ouyang, Dean A Fergusson, Tracy McArdle, Claudio Martin, Deborah Cook, Ian D Graham, Steven Hawken, Colin J L McCartney, Kusum Menon, Raphael Saginur, Andrew Seely, Ian Stiell, Alison Fox-Robichaud, Shane English, John Marshall, Kednapa Thavorn, Monica Taljaard, Lauralyn A McIntyre, Canadian Critical Care Trials Group

**Affiliations:** 1 Clinical Epidemiology Program Ottawa Hospital Research Institute Ottawa, ON Canada; 2 School of Epidemiology and Public Health University of Ottawa Ottawa, ON Canada; 3 Division of Critical Care Medicine, London Health Sciences Centre Western University London, ON Canada; 4 Departments of Medicine, Clinical Epidemiology and Biostatistics St. Joseph’s Healthcare Hamilton McMaster University Hamilton, ON Canada; 5 Department of Emergency Medicine University of Ottawa Ottawa, ON Canada; 6 ICES University of Ottawa Ottawa, ON Canada; 7 Department of Anesthesiology The Ottawa Hospital Ottawa, ON Canada; 8 Children’s Hospital of Eastern Ontario University of Ottawa Ottawa, ON Canada; 9 Department of Medicine, Infectious Diseases The Ottawa Hospital Ottawa, ON Canada; 10 Department of Surgery The Ottawa Hospital Ottawa, ON Canada; 11 Department of Medicine and Thrombosis and Atherosclerosis Research Institute McMaster University and Hamilton Health Sciences Hamilton, ON Canada; 12 Department of Medicine, Division of Critical Care University of Ottawa Ottawa, ON Canada; 13 Department of Surgery St. Michael’s Hospital Toronto, ON Canada

**Keywords:** statistical analysis plan, fluid therapy, normal saline, Ringer’s lactate, cluster randomized trial, pragmatic, comparative effectiveness

## Abstract

**Background:**

Normal saline (NS) and Ringer’s lactate (RL) are the most common crystalloids given to hospitalized patients. Despite concern about possible harm associated with NS (eg, hyperchloremic metabolic acidosis, impaired kidney function, and death), few large multicenter randomized trials focused on critically ill patients have compared these fluids. Uncertainty exists about the effects of these fluids on clinically important outcomes across all hospitalized patients.

**Objective:**

The FLUID trial is a pragmatic, multicenter, 2×2 cluster crossover comparative effectiveness randomized trial that aims to evaluate the effectiveness of a hospital-wide policy that stocks either NS or RL as the main crystalloid fluid in 16 hospitals across Ontario, Canada.

**Methods:**

All hospitalized adult and pediatric patients (anticipated sample size 144,000 patients) with an incident admission to the hospital over the course of each study period will be included. Either NS or RL will be preferentially stocked throughout the hospital for 12 weeks before crossing to the alternate fluid for the subsequent 12 weeks. The primary outcome is a composite of death and hospital readmission within 90 days of hospitalization. Secondary outcomes include death, hospital readmission, dialysis, reoperation, postoperative reintubation, length of hospital stay, emergency department visits, and discharge to a facility other than home. All outcomes will be obtained from health administrative data, eliminating the need for individual case reports. The primary analysis will use cluster-level summaries to estimate cluster-average treatment effects.

**Results:**

The statistical analysis plan has been prepared “a priori” in advance of receipt of the trial data set from ICES and any analyses.

**Conclusions:**

We describe the protocol and statistical analysis plan for the evaluation of primary and secondary outcomes for the FLUID trial.

**Trial Registration:**

ClinicalTrials.gov NCT04512950; https://classic.clinicaltrials.gov/ct2/show/NCT04512950

**International Registered Report Identifier (IRRID):**

DERR1-10.2196/51783

## Introduction

Crystalloid resuscitation fluids, including normal saline (NS) and Ringer’s lactate (RL), are among the most common interventions administered in hospitalized patients [1,2]. These fluids may be used as a life-saving measure to re-establish hemodynamic stability, replace fluid losses, and maintain intravascular volume.

In observational studies, NS has been associated with acute renal injury as compared to RL and other balanced crystalloid fluids, hypothesized to be due to higher chloride concentration and resultant metabolic acidosis that can occur with NS administration [3-5]. However, RL and other balanced crystalloid fluids with buffers have the potential to cause metabolic alkalosis [6,7] and theoretically, cause arrhythmias, tetany, coma, and seizures [8-10]. The lactate in RL may accumulate in the setting of liver failure and may influence clinical diagnoses and clinical decision-making [11-13]. Moreover, RL has a lower osmolarity in comparison to NS; when administered rapidly in large volumes, RL theoretically reduces plasma osmolarity and increases the risk of edema formation [14], which raises potential concern for patients with cerebral edema.

In total, 2 large single-center multiple crossover trials conducted in the intensive care unit (ICU) and the emergency department (ED) among patients who did not require admission to the ICU, found that balanced crystalloid fluids as compared to NS were associated with lower major adverse kidney events at 30 days, which is a composite outcome of mortality, new renal replacement therapy, or persistent renal dysfunction [2,15]. In contrast, 2 large multicenter randomized trials (Balanced Solution versus Saline in Intensive Care Study [BASICS]; Plasma-Lyte 148 versus Saline [PLUS]) [16,17] examined the efficacy of NS as compared to a balanced crystalloid (RL and Plasma-Lyte 148 respectively) on the primary outcome 90-day mortality. Neither of these trials detected differences in 90-day mortality. In BASICS, the mortality rate was 22% versus 21.8%; and in PLUS, mortality was 27.2% versus 26.4% in patients receiving NS and RL, respectively. Renal function did not differ between the fluid groups in either trial, although the PLUS trial was stopped early due to recruitment challenges and insufficient funding during the pandemic. In a systematic review of 35,884 participants in 13 critical care trials published before January 2022, there were no detectable differences in renal function. In low-risk-of-bias trials, there was no significant difference in mortality for the NS as compared to the balanced crystalloid group (28.2% and 27.9% respectively; Relative Risk 0.96, 95% CI 0.91-1.01), nor renal function [15-28]. However, the authors concluded that there is a high probability that balanced crystalloids reduce death since the CIs ranged from a 9% relative reduction to a 1% relative increase in death.

Crystalloid fluids are administered to the majority of patients admitted to the hospital and continued throughout their hospital stay. To address ongoing uncertainty on this topic, and address this evidence gap about fluid administered outside the ED and ICU, we designed a cluster randomized crossover trial. Our objective was to compare a hospital-wide strategy to preferentially stock NS or RL as the main crystalloid resuscitation fluid, aiming to have all admitted patients receive the allocated crystalloid from the time they enter hospital to hospital discharge, evaluating the primary composite outcome of death or readmission to hospital in the first 90 days. The design of the FLUID trial is novel because it involves a pragmatic cluster randomized crossover design, a waiver of patient informed consent to include all hospitalized patients, hospital-based randomization, and the use of routinely collected electronic health administrative data for the description of baseline characteristics and all study outcomes. FLUID will generate data at low risk of bias, providing key evidence about the consequences of crystalloid fluid on clinically important outcomes, and informing patient care and health care policy. It will also provide insight into a novel approach to the conduct of large pragmatic clinical trials.

This manuscript describes the protocol and statistical analysis plan for the evaluation of primary and secondary outcomes for the FLUID trial.

## Methods

### Overview

The protocol and feasibility results for the FLUID pilot trial have been published [29,30]. The 4 pilot sites will be included in the analysis of the large trial as only feasibility outcomes have been previously reported and no major protocol changes were necessary following the pilot trial. The structure of this protocol and statistical analysis plan (version 1.0; March 29, 2023) follows the SPIRIT (Standard Protocol Items: Recommendations for Interventional Trials) Guidelines [31] (see Multimedia Appendix 1 and Table 1) and Guidelines for the Content of Statistical Analysis Plans in Clinical Trials (see Multimedia Appendix 2) [32]. The reporting of the trial results will follow the CONSORT (Consolidated Standards of Reporting Trials) Statement’s extension for Cluster Trials [33] and Randomized Crossover Trials [34], as applicable.

**Table 1:** SPIRIT (Standard Protocol Items: Recommendations for Interventional Trials) table.

Study period										
Preallocation				Postallocation						
Time point		Enrollment (–t_1_)	Allocation (0)	Run-in (1 week)	Period 1 (12 weeks)	Wash-out (2 weeks)	Run-in (1 week)	Period 2 (12 weeks)	Wash-out (2 weeks)	Close-out
**Enrollment**										
	Eligibility screen	✓								
	Allocation		✓							
**Interventions**										
	Fluid A				✓			✓		
	Fluid B				✓			✓		
**Assessments**										
	Demographics	✓								
	Case mix group	✓								
	Type of surgical admission	✓								
	Surgical subgroup	✓								
	Severity of illness	✓								
	Comorbidities	✓								
	Primary outcome									✓
	Secondary outcomes									✓

### Trial Design

The study is a pragmatic, multicenter, 2×2 cluster crossover comparative effectiveness randomized trial. A completed PRECIS-2 table and wheel have been attached as Table S1 and Figure S1 in Multimedia Appendix 3. We defined clusters as hospital sites, allocated to one of two sequences in a 1:1 ratio. The interventions are a hospital-wide strategy of stocking predominantly NS (control) or RL (treatment) throughout the hospital. FLUID is an open-label trial as the cost of blinding the fluids is infeasible and prohibitively costly; this design is in keeping with a real-world evaluation of NS and RL whereby research is integrated into clinical care.

**Figure 1:**
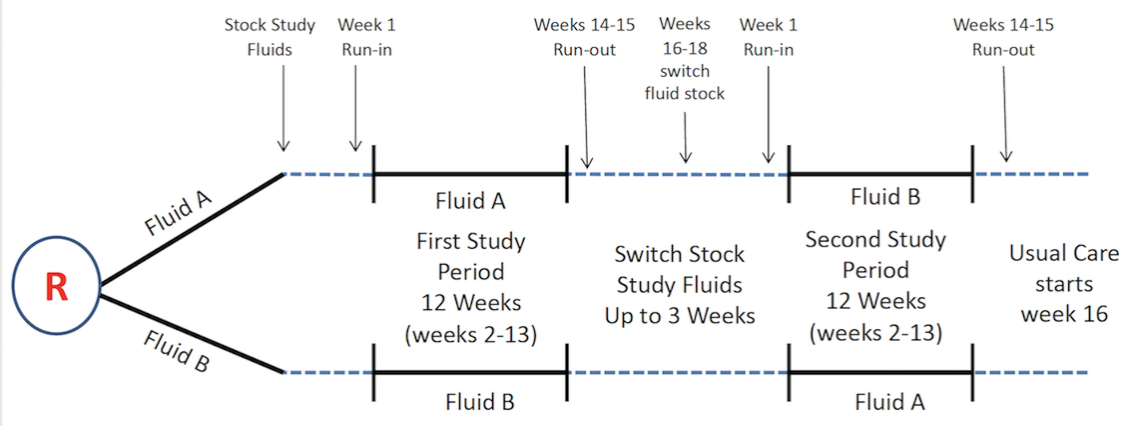
FLUID trial study design.

FLUID has two 15-week study periods. For each study period, week 1 will serve as a run-in, weeks 2-13 (12 weeks) as the study period during which all patients with index admissions to the study hospital will be included for analysis. Weeks 14 and 15 are the run-out period during which time the study fluid remains stocked in the hospital for use by patients admitted during weeks 2-13; however, new patients admitted during weeks 14 and 15 will not contribute to the analysis. After the 2-week run-out period, sites will have 1 week to switch out the study fluid and cross over to the period 2 study fluid. See Figure 1.

### Trial Population

#### Cluster Screening and Eligibility

Inclusion criteria for participating clusters include (1) level II or III ICU capacity [35] and (2) the admission of at least 6000 patients per year to hospital. A list of included study sites will be included in the main trial report.

#### Patient Screening and Eligibility

**Figure 2:**
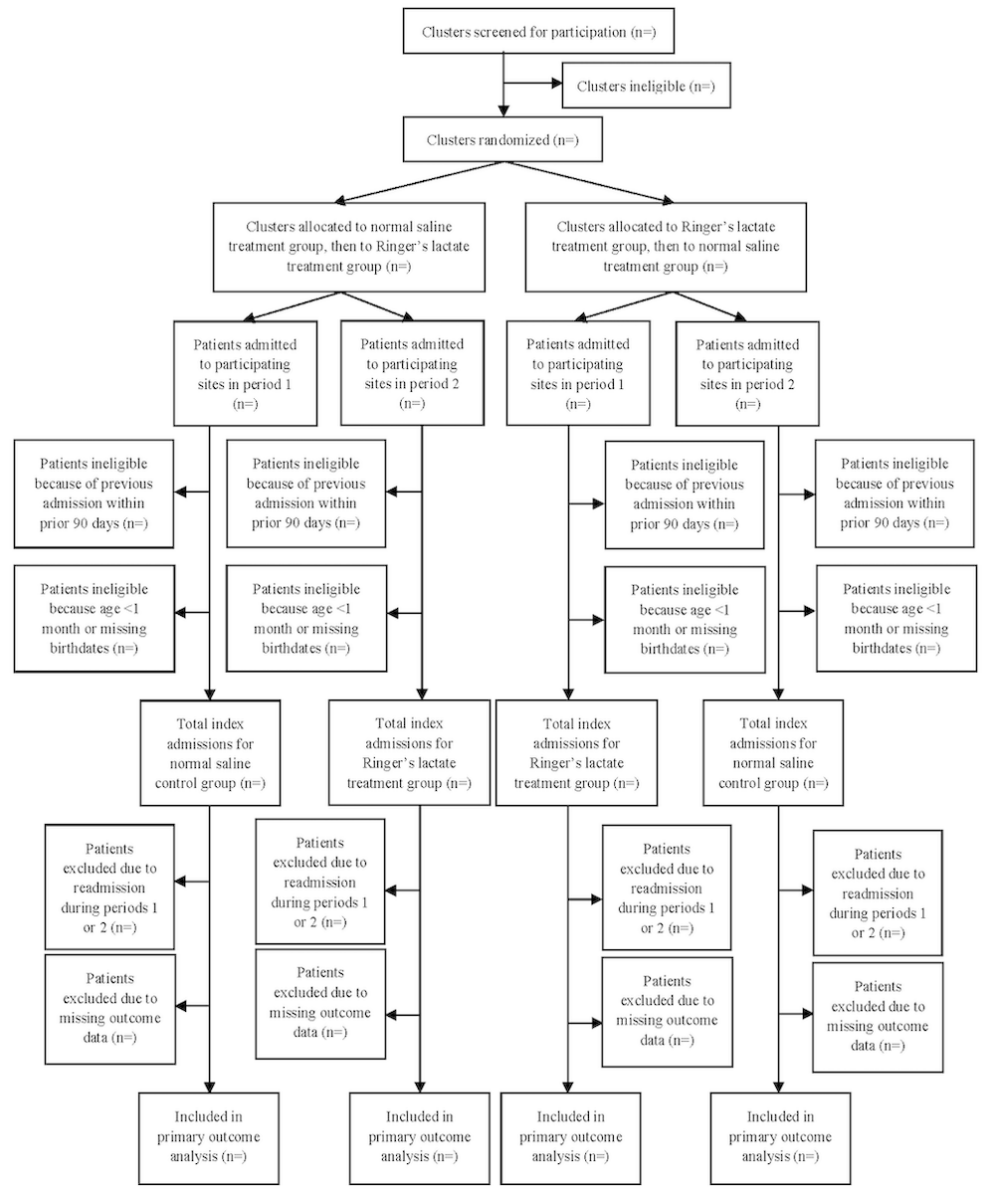
CONSORT (Consolidated Standards of Reporting Trials) flow diagram for participants.

All admitted patients to participating study sites for the first time (index hospital admission) over the duration of the study period will be included under a waiver of informed consent obtained from the research ethics committee; only neonatal patients (age <1 month) will be excluded as RL is not recommended for use in this population [36]. Patients readmitted to the hospital during study periods 1 or 2 and patients admitted during the run-in or run-out study periods will also be excluded. An index admission is defined as a patient admission with no prior hospital admission in the previous 90 days. CONSORT flow diagram of participant recruitment is presented in Figure 2.

#### Randomization

The order of treatment allocation for each hospital site (cluster) will be randomly assigned using computer-generated random numbers, using a permuted block design of length 2, at the coordinating center. The allocation sequence will be prepared by a statistician not familiar with the participating sites, which will be randomized in a 1:1 ratio. Half of the sites will be allocated to sequence 1 and begin the trial with NS as the control, while the other half will be allocated to sequence 2 and begin the trial with RL as the treatment. Each site will cross over to the other fluid after the 12-week study period. To control for period effects, while balancing logistical considerations, randomization will proceed in batches of 4 sites. The allocation sequence will be maintained on a password-protected computer and concealed from investigators until the next batch of sites have been enrolled and are ready to receive their allocation.

### Approach to Safety

No serious adverse events were reported to the coordinating center during the 4-center pilot trial. A Data and Safety Monitoring Committee conducted a review of the data from the 4-center pilot trial, reporting no reason to suspect harm resulting from either fluid. For the main trial, participating hospitals monitor serious adverse events via routinely scheduled meetings of safety committees or morbidity and mortality rounds. Site Principal Investigators report to the coordinating center any serious adverse event occurrence related to study fluid administration.

### Sample Size

A total of 16 hospital sites (including the 4 pilot sites) will yield data on approximately 144,000 hospital admissions. It required 12 sites in a 2-period cluster crossover trial over 6 months to achieve 80% power with a 2-sided α of 0.05 to detect a minimum clinically important absolute difference of 1% in the primary outcome in favor of RL, assuming a proportion of 0.16 in the NS condition, a within-period intracluster correlation coefficient of 0.006, a cluster autocorrelation coefficient of 0.95, and an average of 1500 patients per hospital per month [37]. These estimates were calculated from available routinely collected data for January 1 to December 31, 2013, for all eligible hospitals across the province of Ontario, Canada. Our calculations account for nonadherence based on pilot data (94% adherence NS, 80% RL). We added 2 clusters as an approximate small sample correction [38] and a further 2 to account for cluster size variation [38], for a final sample size of 16 sites.

### Interim Analysis and Stopping Guidance

As a risk to participants in FLUID is minimal, and given the delayed availability of data from the Ontario provincial health administrative databases needed for outcome assessment, no interim analyses are planned in the FLUID trial.

### Adherence and Protocol Deviations

Protocol adherence will not be measured at the individual patient level. Adherence will be measured according to the aggregate use of the study fluid throughout the hospitals using the hospital inventory system. Adherence will be monitored at 2-week intervals over the study period and reported according to each study group across all participating hospitals, combined and according to major fluid user groups in specific settings (ED, surgery, medicine, operating room, ICU, postoperative assessment unit, and obstetrics). Overall adherence will be defined as the total use of the allocated study fluid divided by the total combined use of NS and RL.

### Participant Withdrawal

We will not be collecting individual patient-level data, as all data will be collected through the ICES provincial database. As informed consent is not being sought, only clinically available data are being analyzed, and no unique research data are being collected, participants cannot withdraw from the study.

### Participant Follow-Up

We anticipate near 100% follow-up since all clinical data and outcome measures from participating sites in Ontario will be available at ICES, except for a very small number of patients who leave the province of Ontario within 90 days after enrollment.

### Data Sources

Data will be obtained and housed using Ontario’s population-based health administrative databases at ICES. ICES is an independent, nonprofit research institute whose legal status under Ontario’s health information privacy laws allows the collection and analysis of health care and demographic data. The data sets identified in Table 2 were linked using unique identifiers and analyzed at ICES.

**Table 2:** ICES data sources.

Database	Acronyms
Registered Persons Database	RPDB
Discharge Abstract Database	DAD
Ontario Mental Health Reporting System	OMHRS
National Ambulatory Care Reporting System	NACRS
Ontario Health Insurance Plan	OHIP
Same Day Surgery	SDS
Ontario Drug Benefit	ODP
OHIP lab claims	All OHIP billings have a fee code beginning with “L”
OHIP physician billings	N/A^a^
Home Care Database	OACCAC^b^ HCD
Complex and Continuing Care	CCRS
National Rehabilitation System	NRS
Continuing Care Reporting System	CCRS
Family Health Organization or Family Health Network Capitation	FHO or FHN
Long term care	N/A
National Ambulatory Care Reporting System visits to hospital outpatients clinics and dialysis clinics	NACRS
National Ambulatory Care Reporting System visits to cancer clinics	NACRS
New Drug Funding Plan chemotherapy drugs	NDFP
Assistive Device program	ADP

^a^N/A: not applicable.

^b^OACCAC: Ontario Association of Community Care Access Centres.

### Timing of Final Analysis

All follow-up and data collection for patients at the participating sites will be captured by ICES. The approach to analysis will be supervised by the study statistician and principal investigator; however, the final analysis will be conducted by an independent statistician not involved as a coinvestigator in the FLUID trial.

### Statistical Principles

#### Analytical Framework

All study outcomes will be tested for superiority.

#### CIs and *P* Values

All statistical tests will be 2-sided and performed using a 5% significance level. We will report all CIs as 95% and 2-sided. Results will be expressed as absolute differences produced by analyses described below.

#### Analysis Populations

Our primary analysis will use the intention-to-treat approach and will include all enrolled patients in the treatment groups to which their cluster was randomized at the time of the index hospitalization. Because true fluid exposure status is not recorded in administrative databases, all admitted patients will be included in the analysis, regardless of actual fluid administration.

#### Outcome Definitions

##### Primary Outcome

The primary outcome is a composite of death or readmission to the hospital within 90 days of the index hospitalization. All-cause death will be obtained at a patient level using the Registered Persons Database, while hospital readmission will be measured from Discharge Abstract Database (DAD). Cluster randomized trials can address 2 types of estimands: the participant-average or the cluster-average treatment effect [39]. The former estimates the effect of an intervention for an average participant, whereas the latter estimates the effect for an average cluster. In the FLUID trial, intervention exposure will be unknown at the individual patient-level and the primary study objective is to inform a decision by the health system stakeholder; thus, the primary estimand in the FLUID trial is the cluster-average treatment effect. It is known that cluster-average and participant-average treatment effects will differ when cluster sizes are informative, meaning that either the outcomes or treatment effects vary with cluster size. In the FLUID trial, it is possible that treatment effects will vary between larger or smaller hospitals, and thus, informative cluster sizes cannot be ruled out. The statistical analysis plan was therefore formulated to yield an unbiased estimate for the cluster-average treatment effect in the presence of informative cluster sizes.

##### Secondary Outcomes

The secondary outcomes are 90-day death, hospital readmission within 90 days of the index hospitalization, and requirement for new dialysis within 90 days; and for surgical patients, requirement for reoperation, postoperative reintubation in the postoperative assessment unit, ED visits within 90 days, hospital length of stay, and discharge to facility other than home. All secondary outcomes will be obtained at the patient level from DAD and National Ambulatory Care Reporting System.

##### Tertiary Outcomes

The tertiary outcomes relate to the health economic analyses and include the incremental cost per one death averted and incremental cost per one quality-adjusted life year (QALY) gained.

#### Analysis Methods

##### Descriptive Analyses

Baseline data will be collected and summarized as means and SDs for continuous variables or counts and percentages for categorical variables as shown in Table 3. We will summarize outcome data as cluster-period frequencies and proportions for dichotomous primary and secondary outcomes and medians with IQRs for the length of stay. Outcome data will be reported by the treatment group.

**Table 3:** Baseline characteristics.

Characteristic				Total # index admissions		Normal saline		Ringer’s lactate
Sex, female, n (%)								
Age (years), mean (SD)								
**Age group, n (%)**								
	1 month to 18 years							
	>18 to 65 years							
	>65 to 80 years							
	>80 years							
**Case mix group, n (%)**								
	Medicine							
	Surgery							
	Pregnancy and childbirth							
	Mental health							
**Type of surgical admission, n (%)**								
	Elective							
	Urgent							
	Surgical admission<24 hours							
**Surgical subgroups**								
	General							
	Thoracic							
	Cardiac							
	Vascular							
	Orthopedic							
	Obstetrical							
	Gynecological							
	Ear or Nose or Throat							
	Plastic							
	Urological							
	Neurosurgery							
	Trauma							
	Other							
**Severity of illness, n (%)**								
	Admission to ICU^a^							
	Infection Alone and Infection and Organ Dysfunction							
	Infection Alone and Infection and Organ Dysfunction and ICU admission							
	**Trauma ISS^b^ ≥12, n (%)**							
		Trauma ISS≥12+ICU						
	**Traumatic brain injury, n (%)**							
		Traumatic brain injury+ICU						
**Comorbidities**								
	Elixhauser Comorbidity Score, mean (SD)							
**Elixhauser Comorbidities, n (%)**								
	Diabetes, complicated							
	Hypertension, uncomplicated, and complicated							
	Cardiac arrhythmias							
	Solid tumor without metastasis							
	Fluid and electrolyte disorders							
	Diabetes, uncomplicated							
	Congestive heart failure							
	Metastatic cancer							
	Chronic pulmonary disease							
	Other neurological disorders							
	Peripheral vascular disorders							
	Coagulopathy							
	Valvular disease							
	Obesity							
	Renal failure							
	Paralysis							
	Liver disease							
	Alcohol abuse							
	Pulmonary circulation disorders							
	Depression							
	Deficiency anemia							
	Lymphoma							
	Drug abuse							
	Rheumatoid arthritis or collagen vascular diseases							
	Hypothyroidism							
	Weight loss							
	Psychoses							
	Blood loss anemia							
	Peptic ulcer disease excluding bleeding							
	AIDS or HIV							

^a^ICU: intensive care unit.

^b^ISS: injury severity score.

##### Primary Analyses of the Primary and Secondary Outcomes

All analyses will be conducted according to recommendations for cluster randomized crossover trials, accounting for clustering to ensure correct type I error rates and CIs [38,40]. In the case of a small number of clusters, mixed-effects regression and generalized estimating equation approaches are known to not perform well; analysis for FLUID will, therefore, be performed using cluster-level summaries. This method is known to perform well even with a very small number of clusters. Equal weight will be given to each cluster to obtain unbiased estimates for the cluster-average treatment effect. To implement the cluster-level method, binary outcomes will be expressed as proportions in each cluster-period and the differences (proportion under RL minus proportion under NS will be calculated for each cluster. These differences will then be analyzed using an unweighted linear regression model as described by Turner et al [41]. The t-distribution with degrees of freedom equal to the number of clusters minus 2 will be used. The intervention effect will be expressed as an absolute difference with 95% CI. To improve precision, the primary analysis will account for the following patient risk factors as fixed effect covariates: age, sex, comorbidity index, type of hospital admission (medicine, surgery, pregnancy and childbirth, and mental health), and admissions to ICU, using the 2-stage method recommended by Hayes and Moulton [42]. Patient age will be modeled using a restricted cubic spline to allow for the possibility of a nonlinear association. Mean imputation within hospitals will be used for missing baseline covariates. The same approach and covariates will be used for primary and secondary outcomes. The 2-stage method will be implemented as follows: (1) a multivariable regression model (logistic regression for binary outcomes and linear regression for length of stay) with the baseline characteristics as covariates but excluding the treatment and cluster indicators will be fit to the individual patient data in period 1 and period 2. Length of stay will be log-transformed prior to analysis. We will use each regression model to obtain predicted probabilities for each binary outcome and predicted length of stay for each patient. (2) For binary outcomes, we will calculate the expected number of events by summing the predicted probabilities across all patients in that cluster period; for length of stay, we will obtain the expected mean length of stay by averaging across all patients in that cluster period. (3) We will obtain residuals for binary outcomes by calculating the observed number of events minus the expected number of events divided by the number of patients in the cluster, and for length of stay by calculating the observed mean minus the expected mean. To estimate the treatment effect, the cluster-level method described above will then be applied to the residuals. Estimated intracluster correlation coefficients will also be reported [33]. The data presentation is indicated in Table 4.

**Table 4:** Study outcomes.

		Normal saline (control; n=XXX)	Ringer’s lactate (treatment; n=XXX)	Absolute difference (95% CI)	*P* value
**Primary outcome, n (%)**					
	Composite of death and hospital readmission within 90 days of index admission				
**Secondary outcomes, n (%)**					
	Death within 90 days of index admission				
	Readmission to the hospital within 90 days of index admission				
	New dialysis within 90 days of index admission				
	Requirement for postoperative intubation in the postoperative assessment unit				
	Requirement for reoperation within 90 days of index admission				
	ED^a^ visit within 90 days within 90 days of index admission				
	Hospital length of stay, median (IQR)				
	Discharge to a facility other than home				

^a^ED: emergency department.

##### Subgroup Analyses

The primary outcome will be analyzed within the following subgroups of patients who are at a higher risk for exposure to fluids and with greater risk profiles and or severity of illness than other patients. These include age (<18, 18 to ≤65, 66 to ≤80, and >80 years); sex; type of hospital admission (medical, surgical, pregnancy and childbirth, mental health); elective and urgent or emergent surgical admissions, surgical admissions <24 hours, surgical subgroups (as described in Table 3); admission to ICU, infection alone and infection and organ dysfunction, infection alone and infection and organ dysfunction and ICU admission, trauma with injury severity score (ISS) ≥12, trauma with ISS=12 and ICU admission, traumatic brain injury, traumatic brain injury and ICU admission, and comorbidity index (Elixhauser) [43]. These subgroup analyses will be implemented without covariate adjustment and using the cluster-level summary method as described for the primary and secondary outcomes but applied to subgroup differences within each cluster. Results will be presented using forest plots.

##### Sensitivity Analyses

Testing for carryover effects will not be carried out as the risks of carryover were minimized in the design by allowing for a wash-out period to allow most patients admitted in the first period to complete their hospitalization before the hospital crosses over and by only considering index hospitalizations.

Sensitivity analyses for primary and secondary outcomes will exclude patients who are extremely unlikely to receive the study fluids (direct hospital admissions to psychiatry), those who are otherwise healthy (patients who have vaginal and cesarean births and elective surgical admissions <24 hours), and those who may have been exposed to both types of study fluids (lengths of stay spanning both FLUID study periods).

##### Cost-Effectiveness Analyses

We will conduct a cost-effectiveness analysis of RL and NS from the perspective of the Canadian public health care payer using a hybrid decision tree and Markov models. A decision tree with a 90-day time horizon will be used to determine the effects of each fluid on mortality, requirement for dialysis, and health system costs. Baseline characteristics will be based on the concurrent trial. Surviving patients will enter a Markov component of the model [44] in which outcomes and costs will be modeled in annual cycles for the remainder of the patient’s lifespan. Based on close consultations with coinvestigators, the model structure will incorporate the long-term consequences of fluid therapy; potential health stats may include dialysis-free, dialysis, and death. In each Markov cycle patients could remain in their existing health state, progress to the more severe state, or die. Transition probabilities between health states will be obtained from the concurrent trial and utility values will be derived from the published literature. Direct health care costs, such as physician costs and dialysis costs, will be derived from provincial health administrative data available at ICES and from publicly available sources in Canada.

The model outcomes will include incremental cost per one death averted and incremental cost per one QALY gained. An annual discount rate of 1.5% will be applied to both costs and health outcomes, as recommended by Canada’s economic evaluation guideline [45]. Scenario analyses will be conducted to assess the impact of structural uncertainty and the uncertainty associated with model assumptions on cost-effectiveness results. The model will be fully probabilistic and address parameter uncertainty.

##### Missing Data

The prevalence of missing data is expected to be minimal as outcomes will be obtained from ICES. Nevertheless, missing data for each outcome will be summarized by the treatment group. If substantial missingness is observed (>10%), sensitivity to missing data will be examined under a range of missing-not-at-random scenarios by assuming first that all patients with missing outcomes had the event of interest and then that all patients with missing outcomes did not have the event of interest.

##### Statistical Software

The statistical analyses will be performed with SAS (version 9.4; SAS Institute) and R (R Foundation for Statistical Computing).

#### Dissemination of Findings

Guided by a theory and evidence-informed dissemination planning guide [46], the plan will be guided by the dissemination objective, knowledge of user audiences, dissemination strategies targeted to the audience, and the expertise required to deliver the strategies and required resources. Knowledge user audiences will certainly include health policy leads, health system managers, clinicians, patients and caregivers, and researchers. Depending on the magnitude, strength, and nature of the findings, the dissemination goals could range from increasing awareness of the findings by interested parties to influencing hospital policy and physician prescribing practices at the provincial, national, and international levels. To reach researchers and clinicians, we plan rapid open access publication of our study results in a high-impact journal. We will hold webinars to present and discuss our study results with hospital administrators and clinicians in Ontario. We will reach out to local and provincial social media and traditional media to inform the public, patients, and caregivers about our study findings. If RL is found to be superior to NS, then we will have a particular dissemination focus on health policy and decision-makers to encourage the switch to use these fluids in Ontario hospitals. Finally, we will communicate and present our study findings to the Council of Academic Hospitals of Ontario and plan to host meetings targeted toward health policy decision-makers and high-level hospital administrators (chief executive officers) around the province of Ontario.

### Ethical Considerations

The FLUID Trial was approved at all participating sites through Clinical Trials Ontario (#0778) and the Queensway Carleton Hospital Research Ethics Board (#16-05). Waivers of patient consent were obtained to receive the study intervention and for data collection from ICES, as the risks of study participation are minimal, and requiring informed consent would render the study infeasible.

## Results

At the time of this submission, a total of 7 centers have completed recruitment into FLUID. The statistical analysis plan has been prepared “a priori” in advance of receipt of the trial data set from ICES and any analyses.

## Discussion

The results of this trial will determine whether a hospital-wide policy of stocking predominantly NS versus RL reduces the composite of death and readmission to hospital, as well as other adverse secondary outcomes. We provide a detailed statistical analysis plan which will reduce the risk of data-driven approaches and biased interpretation and increase the transparency of our analyses and results.

There are several challenges related to the design and conduct of the large FLUID trial. These include a risk of study fluid contamination due to a lack of awareness of FLUID, noncompliance to the study protocol, and carryover due to the crossover design. Hence, our team first conducted the FLUID pilot trial in advance of the large trial with the aim of evaluating strategies to address these challenges [29]. In the pilot study, we developed extensive center-specific communication plans for multiple stakeholder groups (eg, chief executive officers, hospital administrators, clinical managers, physicians, nurses, trainees, and logistical services) for implementation in the large trial. To maximize compliance with the study fluid, we implemented (1) an automatic substitution order during the trial study periods for both paper and electronic orders with an override if the treating physician indicates “no substitution” in the physicians’ orders, (2) the hospital ward shelves were stocked with at least 80% study fluid for the duration of the study periods, (3) bright signage prominently placed where NS and RL are stored to help remind nurses about the automatic substitution, and (4) to have the other resuscitation crystalloid fluid available only in small quantities on the clinical shelves. The risk of carryover (effect of study fluid on patients included in study period 1 carrying over to study period 2) is minimized as the vast majority of patients will be different in each study period. The median length of hospital stay is 2 days (10th and 90th percentile 1 and 5.2 days; data derived from FLUID-eligible sites using ICES data); the majority of administered crystalloid resuscitation fluids occur during the first few days of hospital admission; and only index admissions will be included. Furthermore, a 2-week run-out between study period 1 and the end of study period 2 will minimize the occurrence of patients being exposed to 2 different kinds of fluids during the same hospitalization.

### Conclusions

We describe the protocol and statistical analysis plan for the evaluation of primary and secondary outcomes for the FLUID trial. FLUID will determine whether RL as compared with NS reduces death or requirement for hospital readmission by an absolute difference of 1%. In contrast to trials that have generated evidence in specific populations with fluid interventions limited to geographic locations in the hospital (ICU, ED), the results of FLUID will apply broadly to patients who are admitted throughout the hospital. As such, FLUID will provide important evidence-based guidance at the hospital and system level as to what fluids could be predominantly stocked for use throughout the hospital and the associated health care resources required for such supply.

The results of FLUID will help define clinical practice for physicians who care for patients admitted to the hospital regardless of whether differences between the 2 fluid groups in the proposed clinical outcomes are found. The question of the selection of optimal crystalloid fluid is, as evidenced by contemporaneous studies on this theme, an international one, and the results of FLUID will impact hospital management practices around the world. Should FLUID reveal a difference in clinical outcomes for patients, reflected in lives saved and hospital admissions avoided, then one fluid will become the dominant fluid stocked in centers; it may also result in significant savings to the health care system through bulk purchasing of one type of fluid. If no differences are found, then the less expensive crystalloid fluid may become the dominant fluid stocked in centers.

## Appendix

Multimedia Appendix 1 SPIRIT Checklist.

Multimedia Appendix 2 Statistical Analysis Plan (SAP) Checklist.

Multimedia Appendix 3 PRECIS-2 Table and Wheel.

Multimedia Appendix 4 CIHR Peer-review Report.

## Abbreviations

BASICS: Balanced Solution versus Saline in Intensive Care Study
CONSORT: Consolidated Standards of Reporting Trials
DAD: Discharge Abstract Database
ED: emergency department
ICU: intensive care unit
ISS: injury severity score
NS: normal saline
PLUS: Plasma-Lyte 148 versus Saline
QALY: quality-adjusted life year
RL: Ringer’s Lactate
SPIRIT: Standard Protocol Items: Recommendations for Interventional Trials

## Data Availability

The data sets generated or analyzed during this study will not be publicly available as this trial used exclusively health administrative data provided by ICES in the province of Ontario, Canada. The data sets generated and analyzed during the study are not publicly available to protect study participant privacy.
