# Supplementary material for: A Hospital-Wide Open-Label Cluster Crossover Pragmatic Comparative Effectiveness Randomized Trial Comparing Normal Saline to Ringer’s Lactate: Protocol and Statistical Analysis Plan of The FLUID Trial
Source: JMIR Res Protoc. 2023 Oct 6;12:e51783. doi: 10.2196/51783 (PMC10589831; doi:10.2196/51783)

**Multimedia Appendix 3**

**Table S1.** PRECIS-2 scores for trial domains.

|  | Domain | Score | Rationale |
| --- | --- | --- | --- |
| 1 | Eligibility criteria | 5 | All eligible patients admitted to participating study sites were included. Excluded patients would also be ineligible for this treatment in the real world. |
| 2 | Recruitment path | 5 | Patients not recruited for this trial. All eligible patients admitted to the hospital included. |
| 3 | Setting | 4 | Smaller community hospitals are excluded. Participating clusters are required to have level II or III ICU^a^ capacity and an admission of at least 6000 patients per year. |
| 4 | Organization intervention | 5 | No additional resources were provided. No difference in provider expertise or organization of care delivery compared to usual care. |
| 5 | Flexibility of experimental intervention—Delivery | 5 | The delivery of intervention simply involves the substitution of hospital’s fluid for the study fluid and allows for full flexibility, in keeping with usual care. |
| 6 | Flexibility of experimental intervention—Adherence | 5 | Providers are autonomous and given no additional encouragement to adhere to intervention than would be provided in usual care. |
| 7 | Follow-up | 5 | All data is routinely collected from health administrative data. No trial-specific data is being collected. Adherence to intervention is monitored as per inventory. |
| 8 | Primary outcome | 5 | Primary outcome, composite of death, and readmission to hospital were informed and specified by the patient partner. Outcome relevant to patient population, as well as hospitals and providers. |
| 9 | Analysis | 5 | All patients will be included in the analysis according to intention to treat using all available data. |

^a^ICU: intensive care unit.

**Figure S1.** PRECIS-2 wheel.


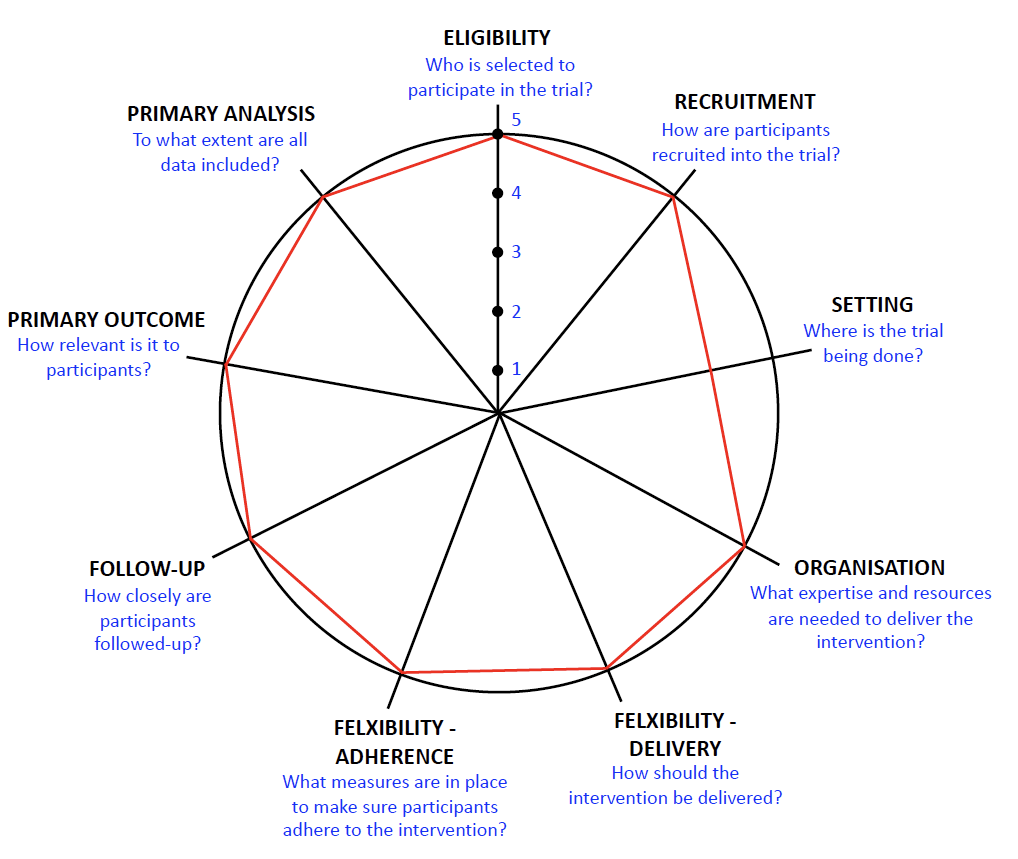

Supplement: Multimedia Appendix 3 [file resprot_v12i1e51783_app3.docx]
