# Supplementary material for: A Hospital-Wide Open-Label Cluster Crossover Pragmatic Comparative Effectiveness Randomized Trial Comparing Normal Saline to Ringer’s Lactate: Protocol and Statistical Analysis Plan of The FLUID Trial
Source: JMIR Res Protoc. 2023 Oct 6;12:e51783. doi: 10.2196/51783 (PMC10589831; doi:10.2196/51783)
Supplement: Multimedia Appendix 4 [file resprot_v12i1e51783_app4.pdf]

**Review Type / Type d'évaluation:**	Reviewer 1 / Évaluateur 1
**Name of Applicant / Nom du chercheur:**	McIntyre, Lauralyn
**Application No. / Numéro de demande:**	399794
**Agency / Agence:**	CIHR/IRSC
**Competition / Concours:**	Project Grant/Subvention Projet
**Committee / Comité:**	Randomized Controlled Trials 2/Essais contrôlés randomisés 2
**Title / Titre:**	Crystalloid FLUID Choices for Resuscitation of Hospitalized Patients: A Pragmatic Comparative Effectiveness Trial (FLUID)

Adjudication Criteria/Critères de sélection

Significance and Impact of the Research/Importance et impact de la recherche: 3.8

Approaches and Methods/Approches et méthodes: 3.5

Expertise, Experience and Resources/Expertise, expérience et ressources: 4.5

Top/Bottom Selection/Groupe supérieur/inférieur

- ○ **Top/Groupe supérieur**
- ◉ **Bottom/Groupe inférieur**

**Review Type / Type d'évaluation:**	Reviewer 1 / Évaluateur 1
**Name of Applicant / Nom du chercheur:**	McIntyre, Lauralyn
**Application No. / Numéro de demande:**	399794
**Agency / Agence:**	CIHR/IRSC
**Competition / Concours:**	Project Grant/Subvention Projet
**Committee / Comité:**	Randomized Controlled Trials 2/Essais contrôlés randomisés 2
**Title / Titre:**	Crystalloid FLUID Choices for Resuscitation of Hospitalized Patients: A Pragmatic Comparative Effectiveness Trial (FLUID)

Summary of Application/Résumé de la demande:

This is a comparative effectiveness trial of two commonly used crystalloid solutions, normal saline versus ringer's lactate for the composite primary outcome of death or hospital readmission within 90 days of index hospitalization. The study is designed using cluster cross-over methodology at 16 hospitals. Each site will be randomized to one solution for 3 months, followed by a one month washout, and then will administer the other solution for 3 months. The investigators are seeking a waiver of informed consent (comparative effectiveness trial). All age ranges, except neonatal patients, are included.

**Review Type / Type d'évaluation:**	Reviewer 1 / Évaluateur 1
**Name of Applicant / Nom du chercheur:**	McIntyre, Lauralyn
**Application No. / Numéro de demande:**	399794
**Agency / Agence:**	CIHR/IRSC
**Competition / Concours:**	Project Grant/Subvention Projet
**Committee / Comité:**	Randomized Controlled Trials 2/Essais contrôlés randomisés 2
**Title / Titre:**	Crystalloid FLUID Choices for Resuscitation of Hospitalized Patients: A Pragmatic Comparative Effectiveness Trial (FLUID)

Strengths and Weaknesses/Forces et faiblesses:

Strengths:

1. Completed pilot study at 4 sites that showed feasibility of protocol.
2. Conducted at both academic and community sites (good for generalizability)
3. Waiver of consent is appropriate for a comparative effectiveness trial of this nature
4. Cluster design is pragmatic and efficient
5. Central randomization
6. Stratification is appropriate
7. Intention to treat

Weaknesses:

1. Specify that this is a human subject trial under certification requirements (pg 2)

2. Reliance on provincial health administrative data through the Institute of Clinical Evaluative Sciences for ALL clinical data collection. While the data may be of high quality (>99% accurate), the number of endpoints and how they are coded are limiting factors in design of this study.

3. Inclusion of all patients admitted to hospital, regardless of reason for admission or unit they were admitted to. While this has some strengths (mainly external validity), the variability in the indication for crystalloid administration, differences in severity of illness and differences in location within the hospital (ie, ICU vs medical unit) will necessitate a large sample size - which they will achieve with this design. However, applying the results of this study clinically will be challenging. Should we conclude that one solution is superior for ALL age ranges and ALL indications?

4. No clinical guidance to try and standardize indications for use of crystalloids (ie, hypotension, acidosis, dehydration). Looking at all indications for replacement together is too broad and misses importance of subgroups where potential effect on survival differ significantly (ie, giving one solution over the other may have a larger impact on sicker patients but no effect for those receiving elective procedures). The investigators state that the effect of differences in baseline characteristics is mitigated by the crossover design.

4. Open label study - without blinding, physician/RN decisions may be altered. For example, they might follow Chloride more closely for those receiving NS. Also, if an clinician prefers NS, then they might use more NS during that phase, and less ringers during the second phase …..this will inflate the differences in exposure.

5. Protocol allows for addition of electrolytes to crystalloid solution as per treating physician. Isn't this a potential confounder? For example, high chloride administration may affect serum bicarbonate levels.

6. Inclusion of adult and paediatric patients but little to no information given to justify inclusion of children using the same protocol. Also, neonates are excluded but the protocol does not specify how 'neonate' is defined (ie, up to 28 days of age).

7. Each epoch lasts 3 months. Patients enrolled if present with index admission for first time in 90 days. This means some patients may end up receiving one or both crystalloid solutions more than once if they are readmitted after 90 days. No mention is made in the protocol/analysis of how this will be managed.

8. Pilot study included 4 sites. This study will add 12 sites. All 16 sites will be used for analysis. Is this permitted? Time bias?

9. data variables (table 3) that are available in the provincial database are suboptimal and likely guided the investigators decisions regarding secondary outcomes. Based on the information in the application, the database is missing important outcomes relevant to this study like edema, weight change, urine output, renal function indices, etc.

**Review Type / Type d'évaluation:**	Reviewer 1 / Évaluateur 1
**Name of Applicant / Nom du chercheur:**	McIntyre, Lauralyn
**Application No. / Numéro de demande:**	399794
**Agency / Agence:**	CIHR/IRSC
**Competition / Concours:**	Project Grant/Subvention Projet
**Committee / Comité:**	Randomized Controlled Trials 2/Essais contrôlés randomisés 2
**Title / Titre:**	Crystalloid FLUID Choices for Resuscitation of Hospitalized Patients: A Pragmatic Comparative Effectiveness Trial (FLUID)

Sex and/or Gender Considerations/Notions de sexe et/ou de genre:

Application states they will enrol patient of both sexes and all genders but does not give further details.

**Review Type / Type d'évaluation:**	Reviewer 2 / Évaluateur 2
**Name of Applicant / Nom du chercheur:**	McIntyre, Lauralyn
**Application No. / Numéro de demande:**	399794
**Agency / Agence:**	CIHR/IRSC
**Competition / Concours:**	Project Grant/Subvention Projet
**Committee / Comité:**	Randomized Controlled Trials 2/Essais contrôlés randomisés 2
**Title / Titre:**	Crystalloid FLUID Choices for Resuscitation of Hospitalized Patients: A Pragmatic Comparative Effectiveness Trial (FLUID)

Adjudication Criteria/Critères de sélection

Significance and Impact of the Research/Importance et impact de la recherche: 4.1

Approaches and Methods/Approches et méthodes: 4.6

Expertise, Experience and Resources/Expertise, expérience et ressources: 4.6

Top/Bottom Selection/Groupe supérieur/inférieur

- (●) **Top/Groupe supérieur**
- ( ) **Bottom/Groupe inférieur**

**Review Type / Type d'évaluation:**	Reviewer 2 / Évaluateur 2
**Name of Applicant / Nom du chercheur:**	McIntyre, Lauralyn
**Application No. / Numéro de demande:**	399794
**Agency / Agence:**	CIHR/IRSC
**Competition / Concours:**	Project Grant/Subvention Projet
**Committee / Comité:**	Randomized Controlled Trials 2/Essais contrôlés randomisés 2
**Title / Titre:**	Crystalloid FLUID Choices for Resuscitation of Hospitalized Patients: A Pragmatic Comparative Effectiveness Trial (FLUID)

Summary of Application/Résumé de la demande:

The applicants propose a pragmatic, comparative effectiveness randomized controlled trial comparing Ringer’s lactate and normal saline. The primary outcome is a composite of death or re-admission to hospital within 90 days of the index hospitalization. Secondary outcomes include death and re-admission described individually, need for dialysis, re-operation or re-intubation post-operatively, emergency department visits, hospital length of stay, discharge disposition, and a health economic analysis.

Design: Cluster cross-over design at 16 hospitals.

Intervention: Hospitals will be randomized to normal saline or Ringers lactate for 3 months, then cross over to the other fluid for the next 3 months after a 1 month washout period. All admitted patients will be included under a waiver of informed consent obtained from the research ethics committee; only neonatal patients will be excluded because Ringer's lactate is not recommended or used in this population. To maximize compliance, the allocated crystalloid study fluid will be stocked throughout the hospital (at least 80%) with only small amounts of the other crystalloid fluid made available.

Outcome data will be obtained at patient-level through the Institute for Clinical Evaluative Sciences. With 16 hospitals the applicants state they will have 80% power to detect an absolute difference of 1% between the study groups at =5%, assuming an estimate of 16% in the normal saline arm, an intra-cluster correlation coefficient of 0.006, and a cluster autocorrelation coefficient of 0.95. These sample size parameters were estimated using routinely collected data from hospitals across Ontario.

Strengths and Weaknesses/Forces et faiblesses:

Strengths:

1. This is a well written application from an experienced group of trialists.
2. The completion of a pilot study demonstrating feasibility is a definite asset
3. The streamlined use of ISIS data is a plus if the data can be obtained reliably

Weaknesses

1.As the applicants point out, there are already 2 large-scale RCT’s evaluating a very similar question, albeit with Plasmalyte, which is similar but not identical to Ringer’s lactate. The applicants point out some differences between these trials and their RCT: FLUID will be performed in all areas of the hospital whereas the other 2 will be performed in certain areas only. The latter will avoid contamination.

2.The rationale for the trial is well presented and the current literature suggests that there it is unlikely there will be a significant difference between the group. The meta-analysis of 16 prior RCT’s (N= approx. 4000 subjects) found there were no significant differences in death (odds ratio (OR) 0.90, 95% confidence interval (CI) 0.69– 1.17) or requirement for renal replacement therapy (OR 1.12, 95% CI 0.80-1.58) for the low chloride as compared to high chloride fluid groups. Based on this, it would be important for the applicants to clearly state their hypothesis. What exactly do they expect to find based on scientific rationale and prior studies? The lack of a clear hypothesis is a limitation.

3.Compliance with study treatment is a major challenge, however I find the applicants have addressed this issue well in the proposal. Their FLUID pilot trial was of major importance in this regard.

5.I agree with the cluster crossover design as it is probably the most efficient way to answer this question.

6. I do have some concerns that data from ISIS will be reliable. Some reassurance on this issue should be given in the grant proposal itself.

**Review Type / Type d'évaluation:**	Reviewer 2 / Évaluateur 2
**Name of Applicant / Nom du chercheur:**	McIntyre, Lauralyn
**Application No. / Numéro de demande:**	399794
**Agency / Agence:**	CIHR/IRSC
**Competition / Concours:**	Project Grant/Subvention Projet
**Committee / Comité:**	Randomized Controlled Trials 2/Essais contrôlés randomisés 2
**Title / Titre:**	Crystalloid FLUID Choices for Resuscitation of Hospitalized Patients: A Pragmatic Comparative Effectiveness Trial (FLUID)

Sex and/or Gender Considerations/Notions de sexe et/ou de genre:

Not specifically addressed in the research proposal

**Review Type / Type d'évaluation:**	Reviewer 3 / Évaluateur 3
**Name of Applicant / Nom du chercheur:**	McIntyre, Lauralyn
**Application No. / Numéro de demande:**	399794
**Agency / Agence:**	CIHR/IRSC
**Competition / Concours:**	Project Grant/Subvention Projet
**Committee / Comité:**	Randomized Controlled Trials 2/Essais contrôlés randomisés 2
**Title / Titre:**	Crystalloid FLUID Choices for Resuscitation of Hospitalized Patients: A Pragmatic Comparative Effectiveness Trial (FLUID)

Adjudication Criteria/Critères de sélection

Significance and Impact of the Research/Importance et impact de la recherche: 4.2

Approaches and Methods/Approches et méthodes: 4.5

Expertise, Experience and Resources/Expertise, expérience et ressources: 4.5

Top/Bottom Selection/Groupe supérieur/inférieur

- (●) **Top/Groupe supérieur**
- ( ) **Bottom/Groupe inférieur**

**Review Type / Type d'évaluation:**	Reviewer 3 / Évaluateur 3
**Name of Applicant / Nom du chercheur:**	McIntyre, Lauralyn
**Application No. / Numéro de demande:**	399794
**Agency / Agence:**	CIHR/IRSC
**Competition / Concours:**	Project Grant/Subvention Projet
**Committee / Comité:**	Randomized Controlled Trials 2/Essais contrôlés randomisés 2
**Title / Titre:**	Crystalloid FLUID Choices for Resuscitation of Hospitalized Patients: A Pragmatic Comparative Effectiveness Trial (FLUID)

Summary of Application/Résumé de la demande:

The trial is a pragmatic cluster cross-over comparative effectiveness randomized trial that will determine if Ringer's lactate as compared to normal saline reduces the primary composite outcome of death or re-admission to hospital within 90 days of the index admission.

**Review Type / Type d'évaluation:**	Reviewer 3 / Évaluateur 3
**Name of Applicant / Nom du chercheur:**	McIntyre, Lauralyn
**Application No. / Numéro de demande:**	399794
**Agency / Agence:**	CIHR/IRSC
**Competition / Concours:**	Project Grant/Subvention Projet
**Committee / Comité:**	Randomized Controlled Trials 2/Essais contrôlés randomisés 2
**Title / Titre:**	Crystalloid FLUID Choices for Resuscitation of Hospitalized Patients: A Pragmatic Comparative Effectiveness Trial (FLUID)

Strengths and Weaknesses/Forces et faiblesses:

A strong research team with appropriate expertise for the study. And the trial is well designed with appropriate analysis methods.

**Review Type / Type d'évaluation:**	Reviewer 3 / Évaluateur 3
**Name of Applicant / Nom du chercheur:**	McIntyre, Lauralyn
**Application No. / Numéro de demande:**	399794
**Agency / Agence:**	CIHR/IRSC
**Competition / Concours:**	Project Grant/Subvention Projet
**Committee / Comité:**	Randomized Controlled Trials 2/Essais contrôlés randomisés 2
**Title / Titre:**	Crystalloid FLUID Choices for Resuscitation of Hospitalized Patients: A Pragmatic Comparative Effectiveness Trial (FLUID)

Sex and/or Gender Considerations/Notions de sexe et/ou de genre:

Gender was well considered.
